# “It was time I could have spent better”—the barriers, enablers, and recommendations for improving access to financial aid when a child has cancer

**DOI:** 10.1007/s00520-025-09347-3

**Published:** 2025-03-15

**Authors:** Megumi Lim, Christine Cashion, Sameera Senanayake, Susanna Cramb, Sanjeewa Kularatna, Natalie Bradford

**Affiliations:** 1 Australian Centre for Health Services Innovation (Aushsi), School of Public Health and Social Work, Faculty of Health, Queensland University of Technology (QUT), Brisbane, QLD Australia; 2 Children’s Health Queensland Hospital and Health Service, South Brisbane, QLD Australia; 3 Duke-NUS Medical School, Health Services and Systems Research, Singapore, Singapore; 4 National Heart Research Institute Singapore, National Heart Centre Singapore, Singapore, Singapore; 5 Viertel Cancer Research Centre, Cancer Council Queensland, Brisbane, QLD Australia; 6 Cancer and Palliative Care Outcomes Centre at Centre for Children’s Health Research, Faculty of Health and School of Nursing, Queensland University of Technology, Brisbane, QLD Australia

**Keywords:** Childhood cancer, Financial aid, Financial assistance, Financial support, Determinants, Barriers and enablers

## Abstract

**Purpose:**

A child’s cancer diagnosis imposes both short-term and long-term stress on families. This study aimed to explore (1) the barriers and enablers in alleviating the financial impacts of a child’s cancer diagnosis and treatment, and (2) areas for improvement in financial aid as suggested by stakeholders.

**Method:**

This qualitative study utilised semi-structured interviews with five hospital social work team members, three charity representatives and eight parents of children treated for cancer. The interviews, which were conducted between April 2023 and January 2024, were analysed using content analysis with a deductive-inductive approach supported by Nvivo Software.

**Results:**

Barriers to mitigating financial impacts included administrative difficulties (e.g. paperwork complexities, strict eligibility criteria and limited support), psychosocial factors (e.g. cognitive burden, social and societal factors), and navigational issues (e.g. poor communication, fragmented support systems). Enablers included streamlined administrative processes, assistance with navigating and applying for financial aid and community support for emotional refuge and respite. Suggested improvements included simplified application forms, offering automatic provision for certain financial aids upon diagnosis, providing infrastructure to support application processes, reallocating funds to increase navigator roles, providing tailored information through a centralised platform, and facilitating connections to parent support groups.

Implications for cancer survivors

The financial stress stemming from the uncertainty of a child’s cancer diagnosis can be overwhelming. Current support systems fall short in effectively mitigating this stress. This research provides empirical evidence for policy changes to enhance support for families, which is crucial to alleviate the multifaceted challenges they face.

**Supplementary Information:**

The online version contains supplementary material available at 10.1007/s00520-025-09347-3.

## Background

Families face significant financial impacts when a child is diagnosed with cancer, with financial stress stemming from disruptions in parental income as a parent is required to take on full-time caregiver responsibilities, and the associated increased direct and indirect expenses [1]. The burdens faced by caregivers of cancer, including financial stress, can negatively impact families' overall mental health, well-being, and health outcomes [2–4]. This financial distress has also been reported as being more overwhelming than physical, social, or emotional difficulties [5–7].

These financial stresses are present across all countries, including those with universal health coverage. For instance, in Australia, 29% of parents of children with cancer reported financial challenges due to direct medical expenses despite the national universal healthcare system [8]. The financial pressures arise from elevated out-of-pocket expenses in the form of direct medical expenses (such as copayments and private health insurance) and direct non-medical costs (such as relocation, childcare, and travel expenses), and indirect costs resulting from lost income [8–12]. Currently available literature indicates that out-of-pocket costs are significantly higher in childhood cancers compared to adult cancers [13]. Several factors contribute to this heightened financial burden. Firstly, children’s cancer treatments often require intensive and aggressive treatment regimens that can lead to life-threatening complications and long-term side effects into survivorship [14–16]. Secondly, because children are highly dependent on their caregivers for medical, personal, and emotional support, parents frequently sacrifice educational and career opportunities, experience involuntary job loss, or choose not to re-enter the workforce to care for their child [14, 17, 18]. The financial impact of these decisions can persist for years after a child has completed treatment. Consequently, in high-income countries, the prevalence of high financial hardship can be as high as 64% [2, 19].

Financial aid is essential in alleviating the economic burden faced by families when a child is diagnosed with cancer. Various forms of financial assistance are available in Australia to address these challenges. These include government support through subsidies and allowances for medication and travel expenses; charitable organisations that provide financial aid for food, accommodation, and cleaning services; and hospital and community programs that help connect families to available resources [8, 10, 20]. However, accessing these supports can be complex, with bureaucratic processes often requiring significant time and effort, adding stress to families already burdened with managing their child’s illness [8]. Additionally, caregivers may find it difficult to discuss their personal finances due to feelings of embarrassment or discomfort [14].

Understanding the barriers to mitigating the financial impacts for families is crucial for policy consideration, even in countries with universal health insurance schemes. A prior scoping review we conducted explored the determinants of effective financial aid in childhood cancers from the perspectives of families and financial aid providers [21]. Among the articles from high-income countries in this review, several barriers were identified, which included knowledge gaps about available help, time-consuming paperwork and processing, difficulties understanding welfare authority communications, insufficient resources for all families, communication challenges between stakeholders, and stigma associated with accessing financial aid [21]. Enablers from high-income countries included dedicated navigators and digital interventions to better identify families in need [21].

To further inform understanding of the financial burden and stress faced by families of children with cancer within the Australian context, we conducted a qualitative study in Queensland, Australia, to gain a deeper understanding of the issues and explore potential solutions. The study aimed to explore (1) the barriers and enablers to accessing financial aid and (2) recommendations for improving the current model of care.

## Methods

### Study design and recruitment

This was a qualitative descriptive study that used semi-structured interviews with key stakeholders. The study was approved by the Queensland Children’s Hospital Human Research Ethics Committee (HREC/22/QCHQ/91661), with informed consent obtained from all participants, who were compensated with coffee vouchers for social workers and $50 Coles or Woolworths vouchers for parents. We structured this report according to the Consolidated Criteria for Reporting Qualitative Research (COREQ) checklist (Supplementary Table 1).

Our study participants were:

(i) Parents of children (< 18 years) who have had a cancer diagnosis (any type) in Queensland, Australia
(ii) Professionals who offer support
  - Oncology social work team members (which included social workers and welfare officers) at the Queensland Children’s Hospital who provide information about financial assistance (QCH)
  - Charity representatives that support families of childhood cancers in Queensland or throughout Australia

A convenience sample of participants who met the inclusion and exclusion criteria (Supplementary Table 2) was recruited. Efforts were made to include individuals from diverse socio-economic backgrounds who were able to discuss their financial situations in relation to their stage in the cancer journey. Study participants were recruited through research nurses in QCH outpatient clinics and existing research team networks, which included the Queensland Paediatric Oncology Support Network (QPOS). Additionally, to enhance representation across different financial circumstances, social workers were actively involved in identifying families with diverse financial backgrounds, and we recruited participants from previous studies where their socio-economic status was known.

## Data collection and analysis

Semi-structured guides (Supplementary Table 3) were used to conduct individual interviews and focus groups to gather perspectives pertaining to (i) the positive and negative experiences with navigating financial assistance schemes, and (ii) suggestions for improvement with regard to the current model of care in financial assistance. The interview guides were informed by the scoping review [21], developed by the research team (ML, SS, SC, NB, SK, CC), and piloted with experienced qualitative researchers (Dr Bridget Abell and Dr Sundresan Naicker) and oncology social work team members (for the parent interview guide) to ensure clarity and organisation of questions.

The interviews were conducted either in person or remotely via Zoom, with only the participants and interviewers present. Consent was obtained on the day of the interviews after ML explained the purpose of the study. Following QCH human research ethic committee (HREC) requirements, participants were not informed of the renumeration until discussions were completed. Interviews were audio-recorded and transcribed verbatim by ML. Field notes were not taken during conversations to maintain engagement; however, debriefs were conducted among the interviewers when they worked together. During these debriefs, they captured key points and emerging themes through structured team discussions, which informed adjustments for future interviews. The interview guide was updated as inductive analysis progressed to explore further salient issues reported in interviews. For example, questions around recommendations for improvement evolved to, “Hypothetically, if you had all the power in the world, what would you change about the current model of care?” Interview summaries were offered to all participants at the end of their interviews. Using verbatim transcripts of the audio recordings, summaries were provided within 4 weeks of the interview. These summaries served the purposes of (i) verifying the data with participants and (ii) familiarising data to help generate initial codes.

Interviews were conducted by ML (a female health economics PhD student with a clinical background in radiotherapy), with assistance from CC (a female children’s cancer research nurse) and NB (a female principal research fellow in children’s cancer and palliative care outcomes). Collectively, the team members have decades of clinical experience with childhood cancers. ML did not have prior relationships with any of the participants before the interviews. CC and NB had prior connections with the social work and welfare team through a previous study. This relationship acted as a double-edged sword; while it facilitated their participation in the current study, it also led to extended discussions driven by concerns related to the study’s findings.

We chose qualitative thematic content analysis for our analysis because the objective was to report on the lived experiences and opinions, rather than uncover hidden sentiments and meanings. We employed a summative content analysis with a deductive-inductive approach, performing line-by-line coding and categorising the codes into themes using Nvivo software [QSR International. (2024). *NVivo (Version 14)*]. The themes were initially informed by our earlier scoping review, which categorised the barriers and enablers into four themes: “accessibility of support”, “delivery of support”, “administration”, and “psychosocial factors” [21]. However, as nuances and contextual variations within the existing themes emerged, themes were refined and recategorised.

The study team (ML, NB, SS, SC, SK) met fortnightly to discuss interview findings, review themes, and address overlaps. The research team collectively assessed and monitored the data during these meetings while recruitment continued. Participant recruitment persisted until data saturation was reached, which was defined as the point when at least two consecutive interviews introduced no new themes or significant insights.

## Results

Seven paediatric oncology social work team members, five charity organisations, and 22 families were invited for interviews. However, two social work team members were unavailable for the focus group interview, two charity organisations declined their involvement, and 14 families were unreachable after expressing interest in the study. In total, we conducted one in-person focus group with five social work team members at Queensland Children’s Hospital, and eleven individual Zoom interviews with three charity representatives and eight parents of children with cancer. During one of the parent interviews, the parent invited the child cancer survivor to contribute to the discussion (parental consent was obtained for the child’s contribution).

All discussions (focus group discussions and interviews) were conducted in English between 26 April 2023 and 12 January 2024, with each session lasting between 43 and 72 min (mean 54 min). The choice to forgo focus groups and only conduct interviews for charity representatives and parent participants was made mainly due to scheduling conflicts. Six interview summaries were requested by participants (social work team, one charity representative, and four parent participants), of which, one parent participant responded with minor additions to the summary after she had escalated her financial struggles to a member of parliament (post-interview). There were no repeat focus groups or interviews, although a follow-up discussion was held with the social work team to address concerns related to the study’s findings.

The participants from all three stakeholder categories were female. The years of experience in paediatric oncology for social work team members and charity representatives ranged from 6 months to more than 5 years. The age of parent participants’ children at diagnosis ranged from 3 weeks to 15 years old. Only one parent participant reported that their child was still on active treatment. Participant characteristics are summarised in Table 1.

**Table 1:** Participant demographics and medical characteristics

Demographics	Social worker team and charity representatives (*n* = 8)
Gender	
Female	8 (100%)
Ethnicity	
Caucasian	8 (100%)
Years of experience in current role	
< 1 year	2 (25%)
1 − 2 years	4 (50%)
> 2 years	2 (25%)
Demographics	Parents (***n*** = 8)
Parental role	
Mothers	8 (100%)
Marital status	
Married	5 (62.5%)
Separated/divorced	3 (37.5%)
Ethnicity	
Caucasian	7 (87.5%)
Indigenous Australian	1 (12.5%)
Employment	
Education (primary/secondary teaching)	2 (25%)
Academia (research/higher education)	2 (25%)
Business owner	2 (25%)
Healthcare worker	1 (12.5%)
Corporate/administration	1 (12.5%)
Area represented (km from Brisbane’s CBD)	
Metropolitan (< 50 km)	4 (50%)
Inner regional (50–100 km)	3 (37.5%)
Outer regional (> 100 km)	1 (12.5%)
Medical characteristics	Children (***n*** = 9)
Age of child at diagnosis	
< 2 years	3
2–10 years	2
10–15 years	4
Cancer type	
Leukaemias and lymphomas	4
Solid tumours (including brain)	5
Time since diagnosis	
< 2 years	3
2–5 years	4
> 5 years	2

**Fig. 1:**
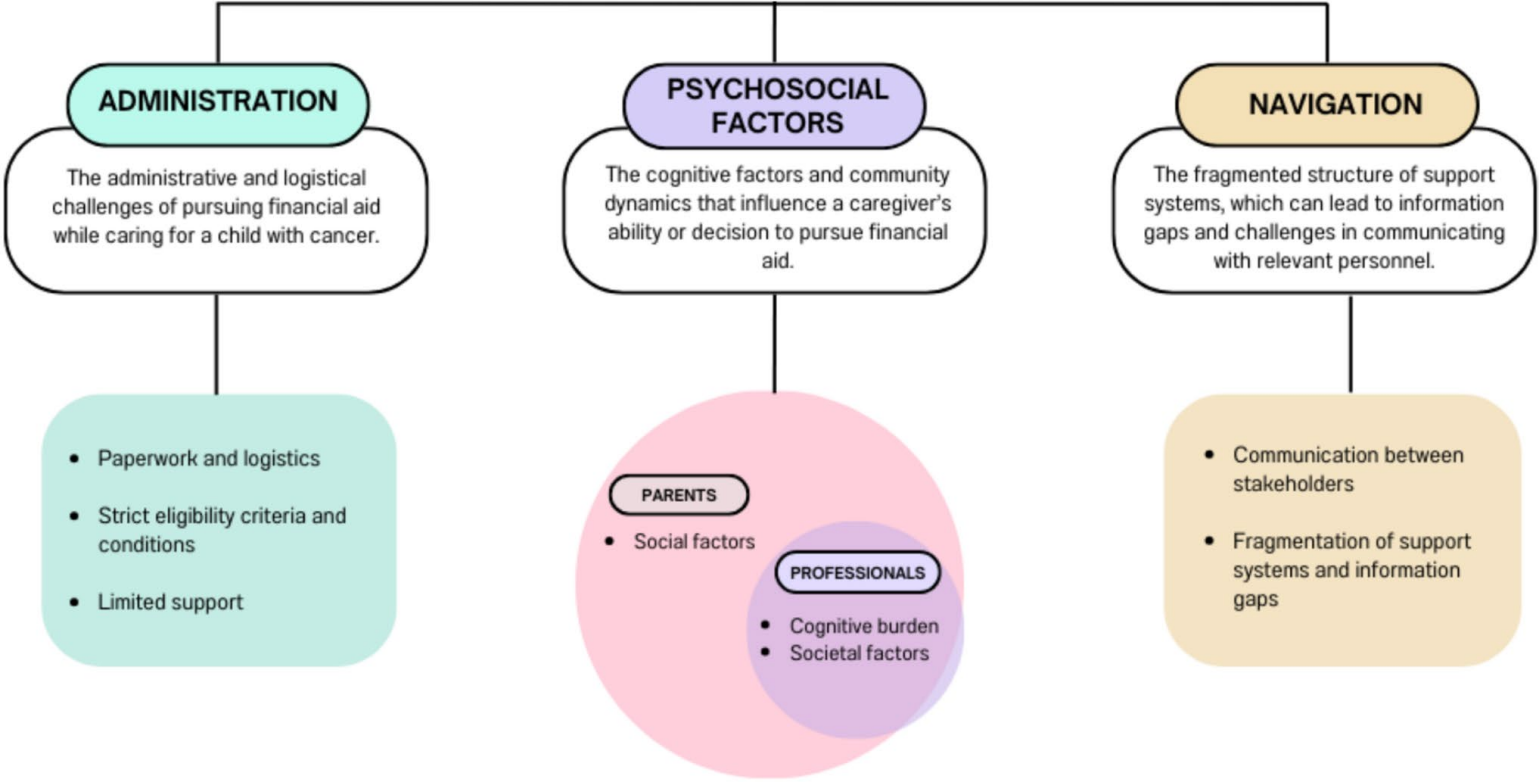
Thematic map

Data saturation was achieved within each participant group type, with the views of the social work and welfare team closely aligned with that of the charity representatives (and were grouped as professional participants), while parents shared similar perspectives. Despite differing roles, the professional participants and parent participants demonstrated significant empathy toward each other’s challenges. The viewpoints from all stakeholders were collectively organised into three main thematic categories (administration, psychosocial factors, and navigation) and eight sub-themes. Each are described, supported by verbatim quotes. The main themes are outlined in Fig. 1, illustrating the overlap of subthemes between parent participants and professional participants (charity representatives, social workers, and welfare officers).

### Theme 1: Administration

#### Sub-theme 1A: Paperwork complexity and logistical constraints

##### Barriers

One common issue raised by all three stakeholder categories was the complexity of paperwork. Although the need for some complexity in paperwork to identify genuine need was acknowledged (parent 6), preparing the necessary documentation was reported as logistically difficult. Some of the logistical difficulties included the need for printed forms while a child is hospitalised (parents 2, 5), keeping a log of every drive to and from the hospital, requiring months’ worth of receipts to be kept for claims (social work participant 4; parent 7), the need to ask for referral letters from their General Practitioner and other allied health professionals every few months (social work participant 4; parents 2, 6), and having to provide tax statements (parent 6). Completing paperwork or going to a government office (parent 7) was described as arduous and time-consuming. This was especially so when there were very young children who were still nursing (parent 6) or when the child was hospitalised, connected to several intravenous lines and the parent could not leave the child’s bedside (parent 2).

As one child and other participants described:

*my mom was like, “oh, I don't really want to leave. Because you could just die at any point in time” … And a lot of parents are like that. I would have been okay to be left (alone) because I was 13, but a lot of these kids are like two or three, and when their parent goes, that makes it even more stressful to them, while being in a hospital with strange things happening to them, while being in pain. – child cancer survivor of parent 5*

*Am I telling… a single parent here… that they have to drive around the local neighbourhood centres to pick up food parcels, or… (with rent), expecting parents who are here bedside with a very sick child to be able to go to look at open homes for rental and apply? – social work participant 4, 2 years in current role.*

For these families, time was a scarce resource, and the difficulty was exacerbated by time limits placed on financial aid applications and the expectation on families to be self-motivated to chase up on applications and reach for help (social work participant 3, 4; parent 1). On top of these logistical difficulties, the format of the questions in application forms (particularly around function and if the disease is terminal) was reported to be overwhelming, confronting, and traumatic to answer (social work participant 4; charity representative 2; parents 1, 8). Consequently, the level of difficulty of questions and length of forms has put some families off from making the application in the first place (social work participant 3).

##### Enablers

With some charity and government applications having moved to an online platform, the need to make phone calls for information has been reduced, and the information families need to provide has been streamlined. This has facilitated easier and quicker applications, and increased speed of responsiveness (parent 3) leading to faster turnaround times, which families have preferred even if the financial amounts given out were smaller (charity representative 3, social work participant 2, 4).

*(Charity organisation - redacted) have an online access portal where they make an account, we approve it and then they can just access the support themselves, which is really great… I think people prefer to cut out to asking someone for help in and be able to go directly to the organisation. – social work participant 4, 2 years in current role.*

##### Areas of improvement

As an area of improvement, some of the parent participants (parents 5, 7, 8) strongly felt that some supports, such as the health cards for subsidised medications and free parking vouchers, should be automatically given with a cancer diagnosis without having to fill out long application forms, especially for schemes that are granted to all Australian citizens and permanent residents.

*we say this in our parent support group. Now when you get your (redacted) bag… In that… we should not be given application forms for funding. It should just be, “Here's your healthcare card. Here's your parking concession card…” Because… when you're in there, it's overwhelming trying to fill out more paperwork and then also physically, especially as a single parent, to just try and sometimes to get half an hour, where my child is comfortable and safe and I can leave her to go to a government office… it can take months to find that time. It's just very difficult. Very, very difficult. – parent 7, child diagnosed at 11 years old.*

Other suggestions included providing printers and high-speed Internet in the wards to help families complete their paperwork (parent 5) and assistance in caring for the sick child so that parents can focus on the applications (parent 8).

#### Sub-theme 1B: Strict eligibility criteria and conditions

##### Barriers

Social work and parent participants both struggled with eligibility criteria around many of the financial aid schemes, such as circumstances where a parent was encouraged to separate from their spouse or to file for separation while the child was on treatment, or had become ineligible for support because they had a Go-Fund-Me [a personal fundraising platform] page.

*(government office) said, “well, if you declare that you were separated then you would be eligible for funding” … at this rate, we're going to end up bloody separated because neither of us is coping with how this is going – parent 1, child diagnosed at 7 years old.*

As one social worker described:

*I had to tell someone recently a few weeks ago, we had arranged for a funeral invoice to be sent to me for support through (organisation). I’d told the family we'll be applying for this; this is what you're eligible for, and I was certain that they didn't have a Go-Fund-Me. And then they went to send off the invoice and then the charity came back to me and… copied and pasted me the link and said, “Is this her? This is her Go-Fund-Me”. Unfortunately, a family friend opened it the day following her death to support with funeral costs, so that was terrible. – social work participant 4, 2 years in current role.*

While ensuring that identifying genuine need was important from all participants, parent participants (parents 1, 4, 5) felt there was no transparency or consistency around eligibility criteria, nor was there consideration for the holistic wellbeing of families beyond income statements. Families who were not in financial need before their child’s cancer diagnosis found it challenging to access financial aid (parents 1, 4, 5).

*(the oncologist) said, “Oh, there's people worse off than you” …… I think if you're not already on welfare, or considered from a low-income family, there's this assumption that you're okay. – parent 4, 2 children diagnosed at 10 and 12 years old.*

##### Enablers

Organisations that recognised that families become financially needy no matter how well off they used to be were praised for not requiring financial statements to be eligible for financial aid.

##### Areas of improvement

Improving transparency and revising means-testing procedures were identified as an area needing enhancement, although acknowledging the difficulty of balancing the relaxation of eligibility criteria with accurately assessing genuine need.

#### Sub-theme 1C: Limited support available

##### Barriers

Despite the complexities of paperwork that families need to navigate and spend time on, the supports available do not come close to replacing an income (social work participant 2; parents 5 and 7). Professional participants identified two main reasons for this. Firstly, charities that offer financial aid to families are reliant on donations, and have struggled to sufficiently meet needs, especially in light of inflation in recent years (charity representative 3; social work participants 1, 3, 5). Secondly, the predominant amount of childhood cancer organisations are research foundations that raise money (and access government funding) to find a cure, with less organisations that provide financial support for families in need (charity representative 2).

*my biggest frustration is that I know that next year I probably won't have as much money (to give out) as I've had this year. – charity representative 3, 2 years in organisation.*

##### Enablers

However, social work participants with extensive experience recognised that there has been much more generosity and recognition of need toward children’s cancers compared to adult cancers or other disease areas.

##### Areas of improvement

It was suggested that the federal government should bear the responsibility of providing support to compensate for families’ loss of income, rather than leaving them at risk of losing their homes (parent 7).

When asked if financial planning services would be beneficial, the social work team noted that such services were available in the community. However, parent participants appeared less interested in knowing upfront costs. One parent participant shared their perspective on why.

people maybe don't need to hear that they're going to be poor and it's going to be really f***ing hard. It's like the last thing that they need to hear. I’m just being blunt. – parent 1, child diagnosed at 7 years old

### Theme 2: Psychosocial factors

#### Sub-theme 2A: Cognitive burden

##### Barriers

The shock families experienced upon learning of their child’s cancer diagnosis have often left many of them unable to process any information. Yet, despite being overwhelmed with the amount of information and the unpredictable nature of the disease, they were expected to make immediate clinical decisions (charity representative 1), which might have caused parents to question whether they were making the correct decision. Parent participants reported being sleep deprived (parent 2), feeling emotionally and mentally drained (parents 4, 5), and experiencing “brain fog” where they could not even remember their own thoughts or questions (parents 5, 8). Consequently, some families did not have the mental capacity to think about their finances (parent 7) and may need months before they were able to start applying for financial assistance (such as health cards for subsidised medications). Of concern, there were instances where parents were not able to backdate applications (social work participants 3, 4, 5), nor the energy to follow up with social workers (parent 6) or the progress of applications (parents 2, 7).

*trying to deal with cancer and parenting is a lot…… You as a family are in that situation and you're learning so much new information, you're fully in survival mode. You're wondering if your kid’s going to survive, you're trying to deal with the side effects. Just being told something or offered something once, is difficult to get, let alone remember or to follow up on. – parent 6, child diagnosed at 3 weeks old*

Some of the younger parents, who are technologically savvy and resourceful (charity representative 2; social work participant 4), had sought out smaller charities for support, which benefitted other families and social workers they inform. On the other hand, some other families had found the combined intensity of the financial and emotional impacts debilitating (charity representative 2; parent 1), especially when it was compounded with pre-existing mental health issues (parent 2).

*my husband wasn't coping…… My husband's barely getting out of bed or barely getting into the shower and getting himself moving, and he's hardly working. And like no support for our other kid. And I can't get support. – parent 1, child diagnosed at 7 years old.*

##### Enablers

Advice to other families going through the journey and experiencing “brain fog” was to have a notebook on hand for easy access to information that has been told, and for recalling important questions (parents 5, 8). To overcome the exhaustion and debilitating cognitive burden, one parent participant (parent 2) recommended that families should delegate tasks (such as form applications) to friends where possible, in order to get some respite. Other parent participants reported that they had only received financial aid because their social worker (parents 2, 7) or a friend (parents 5, 7) had proactively applied on their behalf.

*a mum who I became really good friends with said, “you know, (charity representative’s name - redacted), I said no to the social workers all the time, because we've always managed… But when (child) finished her treatment, we really could have done with the holiday, and we'd used all our money”. – charity representative 1*

Respite services, including holidays, cleaning services, childcare, and even frozen food, were also beneficial for overwhelmed families and something many families desired (parents 2, 8). Unfortunately, these services have not always been accessible to many families.

##### Areas of improvement

Areas of improvement within this sub-theme included improving the presentation of information for families, formatting application forms to reduce the cognitive burden, enhancing support for financial aid applications through clear step-by-step instructions (parent 5), and by providing a navigator (parent 4).

*looking at a lot of the forms, the way that they're presented, it's very intense. You fit a lot to a page and psychologically, it can be very overwhelming when you're already in a very overwhelmed state, so I don't know whether a simplified version of forms or even just formatted differently to allow some breathing space…… I think I would prefer even like a longer document, formatted in a way that is less mentally overwhelming, if that makes sense. – parent 6, child diagnosed at 3 weeks old*

*like with first aid, they have the ABCD and it's very clear, very basic. Because when people are in an accident, they go into shock, and they stop thinking. It's the exact same thing, except this is long-term... As time goes on, your brain gets more overwhelmed. – parent 5, child diagnosed at 13 years old.*

#### Sub-theme 2B: Social factors

##### Barriers

Delegating tasks to friends and family, when faced with cognitive burdens, has not always been a viable option for families. Several parent participants mentioned difficulties in getting the necessary help from their families, such as caring for their other healthy children or assistance with paperwork. It was also not uncommon for families to become isolated because of their child’s cancer (charity representative 3). Parent participants experienced various forms of social isolation: some faced strained family relationships as extended family members preferred visiting only the immunocompromised child (parent 2), while others encountered unhelpful comments from friends regarding the cause of their child's cancer (parent 5). Additionally, some parents experienced social withdrawal from friends who felt emotionally burdened (parent 5), while others lacked the emotional capacity to engage with others due to the challenges they were facing (parents 2 and 3).

*I just felt like I've spent so much time trying to find options… because this is everything that we've worked for (for) 20 years. This is all our savings. This is all we have… still didn't qualify. We have no family in Queensland, we had no friends and nobody that we could get to help us… we've been through years’ worth of savings – parent 1, child diagnosed at 7 years old.*

##### Enablers

Connecting with other families of childhood cancers provided emotional refuge for most parents, though one parent (parent 1) found the parent support group forum unhelpful due to a lack of reliable advice.

*on parent forums, some people will ask a question about, “How do you get a case worker?” and other parents would go, “You go through the hospital” and in the next, “it’s hopeless.” Everyone's just kind of going in circles. – parent 1, child diagnosed at 7 years old.*

Recognising the isolation and emotional toll on families, one of the charities has included psychological counselling in the support services it offers (charity representative 1).

##### Areas of improvement

Recognising that parents often are preoccupied with looking after their child and may not feel inclined to interact with other parents, suggestions to facilitate parent connections included establishing a parent corner in the ward (parent 8) and placing a quick response (QR) code at each bedside to direct families to the QPOS village (parent 7). For the latter, the parent favoured prompt implementation and was planning discussions with the hospital to facilitate this.

#### Sub-theme 2C: Societal factors

##### Barriers

Although families do not want to put financial woes onto their children (parent 3), it is not uncommon for families to reject financial aid early in the cancer journey. Some reasons that families did not seek help early included feelings of shame in admitting they could not pay rent (charity representative 3), stigmatisation and lack of empathy stemming from the perception that families abuse the system to avoid working (parent 4; social work participant 4). Also reported were a sense of guilt toward other families who might need aid more (parent 4; social work participant 4; charity representative 3), and concerns about being seen as a nuisance to social workers (parent 3). This sense of guilt has prevented families from even wanting to discuss their finances (parent 4), resulting in families only seeking help further into treatment when they felt the impacts (social work participant 5) or when their situation became dire (charity representative 3). This sentiment was palpable in our study when one of our parent participants (parent 4) expressed hesitation about participating, as she felt she was financially fortunate compared to other families and undeserving of sharing her opinion.

##### Areas of improvement

The suggestion for improvement was to have an emergency case worker to address instances of financial crises, such as the risk of homelessness due to unpaid rent.

(parent 2; charity representative 3).

*people have got to be prepared to talk about it when the problem arises, but we've got to be there quickly to solve it before people are at risk of homelessness – charity representative 3, 2 years in current role.*

### Theme 3: Navigation

#### Sub-theme 3A: communication between stakeholders

##### Barriers

Contacting the appropriate personnel for information and assistance with application forms was a frequent source of frustration and distress for both parent participants and the social work team, due to the long wait times on hold and the need to repeat their traumatic circumstances with different people.

*Quite frankly I should have just not bothered… Like if you added it up cumulatively, it would have been weeks of my life trying to find options to try and make it better or easier, or find some solutions, or on the phone or on hold, or trying to get answers…… I just wish that I'd never bothered because it was time that I probably could have just better spent with my kid…… it is time I can't get back with him. I'm just lucky that I still have him… but you know, if we didn't have that outcome… – parent 1, child diagnosed at 7 years old*

Administrative processes were also often lengthy and demoralising, as several participants (parents 1, 2, 8; social work participant 2; charity representatives 2, 3) noted, with paperwork often taking months to be approved. When administrative mistakes occurred, the rectification process further exhausted families (parents 1, 6, 7, 8).

*It took them three months to decide that they needed to clarify some of my information, and then another four months to reject me. – parent 1, child diagnosed at 7 years old*

##### Enablers

Advocacy, whether carried out by families themselves or through a general practitioner or social worker, was seen as crucial for improving administrative processes (social work participant 3) and addressing families’ needs (parents 2, 3, 5). Social work participants indicated that having a designated contact person in government offices has helped address missed applications (social work participant 3), and having welfare officers in their team (funded by a charity) assisted them in managing their workload effectively, staying updated on changing eligibility criteria, connecting families to support services, and adhering to their model of care (social work participant 1).

*it's (been) really, really hard for a while but now we've got another worker on board… and the difference that's made is enormous. So I think since then, we've really been able to focus on some people before I just couldn't get to in time; they were going on sometimes 5 weeks before I even got to speak with them, which I felt terrible about. But now I think we're really sticking to our model of care better, focusing more on and taking the time with families, rather than trying to get through a bunch of 6 phone calls in a day because you don't even have time to go to the ward. – social work participant 4, 2 years in current role.*

The importance of having a well-staffed welfare team was raised, as navigators are often better equipped emotionally to manage these processes (charity representative 2). Additionally, the social work team have also frequently served as the sole source of financial aid information for many families (parents 2, 3, 7).

*I do believe that the role of navigators and nurse consultants that actually stay with the patient from diagnosis, until they're at least in a stable and settled situation, is essential…… I had a client say to me this morning, “I don't understand how you all do this. How do you keep up with it all?” And I go, “Because we're not going through what you're going through. We're in a place of being able to manage our time and know what our day holds. You don't have that… cut yourself a little bit of slack, you're in another zone”. – charity representative 2, 18 months in current role.*

##### Areas of improvement

It was suggested that the information should be available in a range of languages (charity representative 3).

#### Sub-theme 3B: Fragmentation of support systems and information gaps

##### Barriers

When a child is newly diagnosed with cancer, families receive a bag containing several pamphlets from charities offering financial support. However, since the same bag is given regardless of the child’s specific cancer type, families must sift through the information to find what is relevant. This task of navigation was overwhelming for some already exhausted families, who then had to make phone calls to charities did not qualify for (parents 7, 8). It is unfortunate that the fragmented nature of the welfare system has meant that the responsibility has fallen on families to follow up and seek help, despite their limited time and mental bandwidth. Unfortunately, the limited time and mental bandwidth social workers themselves face when providing support to families can mean that some families are lost to follow up (charity representative 1, 2; parents 4, 5, 6, 7, 8).

Families also experienced shock when the child was discharged from the hospital, and had to navigate the health system without support (parent 8). This lack of navigational support after discharge, unfortunately, compounded the financial turmoil, which continued even at the completion of acute cancer treatment. Many parent participants reported that as their child entered survivorship, they have faced ongoing long-term side effects necessitating referrals to various medical specialties, and carried the burden of the potential for secondary cancers. The continuous medical follow-ups after returning home have left these families both emotionally and financially exhausted (parents 4, 5), but with significantly less support and access to financial assistance.

##### Enablers

Several participants (social work participant 4; parents 1, 4, 5, 8) mentioned that connecting with other families was helpful for finding out information about available financial aid resources and their eligibility. This access to one financial aid resources sometimes also created a spillover effect, which lead families to discovering other resources (parents 2, 8).

##### Areas of improvement

One suggested improvement was to create an integrated one-stop-shop, such as a catalogue or mobile application, to provide tailored information specific to the child’s disease on a centralised platform (parents 1, 4, 6, 7, 8). This would help families quickly navigate available resources, even while in the hospital ward.

A second suggested improvement for better supporting administrative processes was to reallocate some funding—from government or research funding sources—to increase the number of staff dedicated to navigating and advocating for families (charity representative 1; parents 4, 5). This support is especially crucial during survivorship when children are discharged from the hospital (charity representative 1).

*I would like the hospital to have more people on the ground just checking in on families. Because even when people say they're okay, they're probably not. And you don't want to be a bother to people, but you know, just understanding that they're there for you… you're not a bother, but you feel like you are. – parent 4, 2 children diagnosed at 10 and 12*

## Discussion

This study provided a deeper understanding of barriers and enabler of accessing financial aid that was built upon our earlier scoping review [21]. The scoping review categorised the barriers and enablers into the themes of “accessibility of support”, “delivery of support”, “administration”, and “psychosocial factors” [21]. Our current study identified similar themes, but revealed greater overlap and interaction from other confounding factors. This led to the recategorisation of themes, which were “administration”, “psychosocial factors”, and “navigation”. Additionally, we also asked our participants, who have lived experience or professional expertise in this area, to make recommendations for improvement.

Accessing financial aid when a child has cancer presents numerous challenges for families. The administration of financial aid for families with children diagnosed with cancer is fraught with complexities and logistical challenges. Our participants highlighted the burden of navigating support systems and dealing with the overwhelming and traumatic application processes. The paperwork complexity is compounded by the logistical difficulty of acquiring necessary documents while managing a child’s treatment. Strict eligibility requirements further complicate access to financial aid, with inconsistent criteria and a lack of transparency adding to the frustration. Limited financial support from charities, often struggling to secure donations, fails to compensate for lost income. Additionally, the cognitive burden of processing a child's cancer diagnosis leaves families mentally and emotionally drained, making it difficult to manage finances and seek aid. This burden is often intensified by fragmented support systems and lack of continuity of care, leading to delays and missed opportunities for financial assistance. Consequently, although finances are one of the top priorities for families, the struggle to secure the needed support has left many discouraged.

Consistent with existing literature, our participants also identified financial strains and psychological distress stemming from the combination of heightened spending and diminished incomes [22]. A recent qualitative study from the USA noted that, unlike adult cancers where medical bills are the primary driver of expenses, caregivers of children with cancer often identified non-medical costs, such as food, accommodation and transportation, as main financial burdens [22]. Similarly, parent participants in our study described that during active treatment, sources of escalated expenses included transportation and parking fees, medication costs, cleaning services, food costs during prolonged hospital stays or visits, rent inflation, and elevated utility bills. Post-treatment, increased financial burdens arose from having to modify the child’s education on top of the ongoing medical costs associated with managing treatment side effects and addressing mental health impacts on family members. The resultant financial hardships include depleted savings and the inability to save for extended periods, difficulties affording basic necessities like food, and the risk of homelessness. It was highlighted that families need enhanced support, both during and after treatment, to alleviate the multifaceted challenges they face.

At first glance, the socio-economic backgrounds of our parent participants might appear relatively homogenous, given their educational qualifications and career histories. However, despite the seemingly privileged profile of our sample, several parents reported experiencing food insecurity and reliance on Centrelink payments, income protection, or other welfare programs. This was an unexpected finding, particularly for those with strong career profiles, such as healthcare workers and teachers. Taking extended leave, reducing work hours, or even leaving the workforce entirely dramatically changed these families’ financial situations, leaving once-stable families vulnerable to financial strain. As highlighted in our results, there is a clear need for improved financial aid models that extend beyond traditional income-based eligibility. These models should be designed to address the evolving needs of families, ensuring that fundamental needs such as food security are met throughout treatment and into survivorship.

We also found the lack of interest in financial planning intriguing, as previous research in adult cancers has suggested that providing upfront information about expected costs can help reduce financial stress [9, 23, 24]. This difference is likely due to the fact that children’s cancers are often more aggressive and unpredictable [25], which not only results in higher out-of-pocket costs [13] but also presents barriers for parents’ maintaining or returning to employment [26]. Many parents scale back their employment or business activities to care for their children during a life stage marked by increased financial dependence as opposed to older adults who may have access to retirement funds, and hence, often rely on income protection and financial aid programs to stay financially afloat [11, 22, 27]. Consequently, recommendations for addressing financial toxicity in paediatric oncology studies generally revolve around overcoming the logistical and navigational challenges of accessing financial aid [22]. This, unfortunately, does not reflect better working conditions or greater employer understanding due to a child’s cancer diagnosis as some of our families reported job losses due to redundancy (parent 6), job sabotage (parent 4), and post-traumatic stress (parent 2).

Evidently, the financial impacts of a child’s cancer diagnosis are significant and can extend beyond the end of treatment, continuing for years into survivorship. With an increasing percentage of childhood cancer patients surviving, survivors and their families must also contend with the possibility of recurrence, which stands at a cumulative incidence of 4.4%, 5.6%, and 6.2% at 10, 15, and 20 years, respectively, conditional on surviving five years post-diagnosis without experiencing a relapse or death [28]. Therefore, greater efforts are needed to prevent families from experiencing severe financial hardships. In this regard, it was encouraging to see that charity representatives, social work participants, and members of the parent support group (QPOS), were actively working to improve processes for enhancing families' financial well-being. Reported enhancements in progress at the time of interviews included the implementation of regular follow-up assessments at 12 weeks, 6 months, and 12 months after diagnosis (social work team), the development of an intervention program for nursing coordinators to support families during their transition from hospital care to normal life (charity representative 1), and the active advocacy for free parking for oncology families (parents 4, 5, 7; charity representative 3).

We acknowledge several limitations in our study. Firstly, the scope of participant selection was restricted to parents for their firsthand experience in navigating financial aid and their insights into how peripheral issues contributed to their challenges. Social workers, welfare officers, and charity representatives were included as key decision-makers who play critical roles in the success or failure in financial aid processes, as highlighted in the scoping review. However, we recognise the contributions of other stakeholders, such as advocates, navigators, nurses, and physicians, whose perspectives could provide a more comprehensive understanding of the financial aid processes and its interaction with clinical care. Secondly, the demographic composition of our participants was largely homogeneous (e.g. predominantly highly educated, white Australian females), and therefore, did not capture the perspectives of minority populations. Despite our best efforts, we faced the challenges of low participation rates which resulted in a relatively small sample size. Although 22 parent participants initially expressed interest and provided their contact details, 14 of them later became unreachable. We were also unable to recruit more charity representatives, as they either did not respond to our invitations or declined participation due to concerns about overburdening their staff. With the small sample size, there is potential for bias in our findings due to the voluntary nature of participation, as individuals with strong opinions may have been more likely to participate. However, despite the small sample of parent participants, the findings identified through rich, in-depth interviews with parent participants were consistent with the insights from the social work team and charity representatives. Continuous data review during team meetings confirmed that no significant new insights emerged data saturation was reached; the research team concluded that the sample size was thus sufficient. While a larger and more diverse sample may have provided additional nuances, the team was confident that the key financial challenges, enablers, and suggested improvements were thoroughly explored within this cohort. Future research would benefit from more targeted recruitment strategies, particularly focusing on underrepresented groups—including families who face challenges such as limited internet access, low literacy, or language barriers—and utilising simplified surveys to mitigate barriers such as time constraints, cognitive fatigue, and emotional stress, to capture a broader range of experiences around financial hardship. Thirdly, we employed a mix of data collection methods due to challenges in coordinating participant schedules. Opting for individual interviews instead of focus groups during the study reduced opportunities for dynamic discussions and the exploration of diverse perspectives. Additionally, variations in data collection methods may have influenced the type of data provided by participants across different categories, not only due to their roles but also due to differences in how questions were framed. However, conducting individual in-depth interviews with parent participants offered greater privacy for sharing sensitive information, minimised social desirability bias, allowed for a more iterative process of data collection and ensured data completeness [29]. Lastly, our study primarily included participants from a single site, which although is the sole paediatric hospital in Queensland serving as a major referral centre for all children diagnosed with cancer across Queensland and northern New South Wales, there may be limited transferability of findings to other settings.

## Conclusion

Our study provided valuable insights into the experiences of families, social workers, and charity representatives within the current model of care, as well as the improvements they would like to see. It underscored the need for more accessible financial support, as the challenge of balancing employment with caregiving responsibilities often exacerbates financial strain. To address these issues, policy reforms should focus on streamlining financial aid processes and improving communication about available support. These findings can inform the development of more effective financial interventions in Australia and other countries with similar systems. However, further research is necessary to explore families’ preferences for financial assistance and to determine the most impactful policy changes.

## Supplementary Information

Below is the link to the electronic supplementary material.

Supplementary file1 (DOCX 27 KB)

## Data Availability

The datasets generated during the study are available from the corresponding author upon reasonable request.
