# Supplementary material for: “It was time I could have spent better”—the barriers, enablers, and recommendations for improving access to financial aid when a child has cancer
Source: Support Care Cancer. 2025 Mar 15;33(4):284. doi: 10.1007/s00520-025-09347-3 (PMC11910398; doi:10.1007/s00520-025-09347-3)
Supplement: Supplementary file 1 — Supplementary file1 (DOCX 27 KB) [file 520_2025_9347_MOESM1_ESM.docx]

### Supplementary table 1: COREQ checklist

| **Topic** | **Item No.** | **Guide Questions/Description** | **Reported on Page No.** |
| --- | --- | --- | --- |
| **Domain 1: Research team and reflexivity** | | | |
| *Personal characteristics* | | | |
| Interviewer/facilitator | 1 | Which author/s conducted the interview or focus group? | 3 |
| Credentials | 2 | What were the researcher’s credentials? E.g. PhD, MD | 1 |
| Occupation | 3 | What was their occupation at the time of the study? | 3 |
| Gender | 4 | Was the researcher male or female? | 3 |
| Experience and training | 5 | What experience or training did the researcher have? | 3, 19 |
| *Relationship with participants* | | | |
| Relationship established | 6 | Was a relationship established prior to study commencement? | 4 |
| Participant knowledge of the interviewer | 7 | What did the participants know about the researcher? E.g. personal goals, reasons for doing the research | 4 |
| Interview characteristics | 8 | What characteristics were reported about the interviewer/facilitator? E.g. bias, assumptions, reasons and interests in the research topic | 3 |
| **Domain 2: Study design** | | |  |
| *Theoretical framework* | | |  |
| Methodological orientation and Theory | 9 | What methodological orientation was stated to underpin the study? e.g. grounded theory, discourse analysis, ethnography, phenomenology, content analysis | 3 |
| *Participant selection* | | | |
| Sampling | 10 | how were participants selected? E.g. purposive, convenience, consecutive, snowball | 3 |
| Method of approach | 11 | How were participants approached? E.g. face-to-face, telephone, mail, email | 3 |
| Sample size | 12 | How many participants were in the study? | 4 |
| Non-participation | 13 | How many people refused to participate or dropped out? Reasons? | 4 |
| *Setting* | | | |
| Setting of data collection | 14 | Where was the data collected? E.g. home, clinic, workplace | 3 |
| Presence of non-participants | 15 | Was anyone else present besides the participants and researchers? | 4 |
| Description of sample | 16 | What are the important characteristics of the sample? E.g. demographic data, date | 5 |
| *Data collection* | | | |
| Interview guide | 17 | Were questions, prompts, guides provided by the authors? Was it pilot tested? | 3 |
| Repeat interviews | 18 | Were repeat interviews carried out? If yes, how many? | 5 |
| Audio/visual recording | 19 | Did the research use audio or visual recording to collect the data? | 4 |
| Field notes | 20 | Were field notes made during and/or after the interview or focus group? | 4 |
| Duration | 21 | What was the duration of the interviews or focus group? | 4 |
| Data saturation | 22 | Was data saturation discussed? | 4 |
| Transcripts returned | 23 | Were transcripts returned to participants for comment and/or correction? | 4 |
| **Domain 3: analysis and findings** | | | |
| *Data analysis* | | | |
| Number of data coders | 24 | How many data coders coded the data? | 4 |
| Description of the coding tree | 25 | Did authors provide a description of the coding tree? | 7 |
| Derivation of themes | 26 | Were themes identified in advanced or derived from the data? | 4 |
| Software | 27 | What software, if applicable, was used to manage the data? | 4 |
| Participant checking | 28 | Did participants provide feedback on the findings? | 4 |
| *Reporting* | | | |
| Quotations presented | 29 | Were participant quotations presented to illustrate the themes/findings? Was each quotation identified? E.g. participant number | Yes |
| Data and findings consistent | 30 | Was there consistency between the data presented and the findings? | Yes |
| Clarity of major themes | 31 | Were major themes clearly presented in the findings? | Yes |
| Clarity of minor themes | 32 | Is there a description of diverse cases or discussion of minor themes? | Yes |

### Supplementary table 2: participant inclusion/exclusion criteria

|  | **Parents participants** | **Charity representatives and social work participants** |
| --- | --- | --- |
| **Inclusion criteria** | - The parent has had a child with cancer within the past 10 years; - The child and parent reside in Australia and received treatment and follow-up care in Australia; - The family had sought financial assistance during the child’s cancer treatment; - The parent is above 18 years of age. | - Australian-based service providers (such as oncologists, nurses, counsellors, medical social workers, welfare officers or hospital administrators); - Members of non-governmental organisations that provide financial assistance |
| **Exclusion criteria** | - The child’s last cancer treatment or follow-up care was more than 10 years ago; - The parent is unable to converse in English; - The parents do not have the mental or physical capacity to provide consent or adequately express their opinions. |  |

### Supplementary table 3: semi-structured interview guides

| Category of questions | Participant stakeholders | | |
| --- | --- | --- | --- |
|  | **Hospital social workers** | **Charity representatives** | **Parents of children with cancer** |
| Background information | Tell me about your role, and how long you’ve been in this role for? | | How did you find out your child had cancer? |
| Experiences with financial impacts and needs | - In your experience, how often do families voice out a need for financial aid? - What sorts of financial challenges have you seen families face? | | - How has your financial situation been impacted/changed since? - Did you seek financial help?   - *Yes/no, why?* - At what stage did you seek financial aid? |
| Barriers of financial aid access | - What are some of the challenges do families face when seeking financial aid?   *Centrelink, healthcare card, travel subsidy, keeping up with claims*   - What are some of the difficulties you have faced in your role? | | - Could you share some of your experiences (both good & bad) around seeking financial aid?   *Difficulty navigating through the system*  *Didn’t have time to address all the paperwork, too fragmented, too much paperwork*   - What were some of the sources of these challenges?   *difficulties talking about money to others, feeling overwhelmed from changes in family life and dynamics, knowing your eligibility for the scheme, the time needed to prepare the necessary documents, having other priorities that took precedence over finances* |
| Enablers of financial aid access | - Have you (and the families you’ve helped) had positive experiences with financial aid providers?   *Yes/no, what makes you feel that way?*  *Having a responsive coordinator from the organisation, having a navigator to help with applications*   - What do you think helped? - What have you found to be most rewarding in your work? | | - From where did you seek information about financial assistance schemes?   - *Who provided information? (welfare/other parents in ward/Facebook)* - What do you think has been most helpful/were there any positive experiences when seeking financial support? - Based on your experiences around finances, what advice would you give to other parents going through this journey? |
|  |  | - What sorts of feedback have families given around their experiences? - How does the organisation decide on what to provide for/eligibility?   *Literature review? Focus group discussions?* |  |
| Areas of improvement | - How do you think current systems can be changed/improved to better support both families and charity organisations?   *What government policy changes could make a difference?*  *Pamphlets for navigating resources after discharge, referrals for social workers outside the hospital* | | - Looking back at your experiences, what do you think could have been improved for you regarding finances (at the beginning, during, and after treatment)? - From your interactions with other families, what do you think other parents (in general) need when seeking financial help? - If you could design your own financial aid model of care, what would you do differently? |
|  | - What do you think would help improve your experience as a social work team member? |  |  |
